# Connectome-Scale Assessments of Functional Connectivity in Children with Primary Monosymptomatic Nocturnal Enuresis

**DOI:** 10.1155/2015/463708

**Published:** 2015-06-09

**Authors:** Du Lei, Jun Ma, Jilei Zhang, Mengxing Wang, Kaihua Zhang, Fuqin Chen, Xueling Suo, Qiyong Gong, Xiaoxia Du

**Affiliations:** ^1^Shanghai Key Laboratory of Magnetic Resonance, Department of Physics, East China Normal University, 3663 North Zhongshan Road, Shanghai 200062, China; ^2^MR Research Center, Department of Radiology, West China Hospital of Sichuan University, Chengdu, Sichuan 610044, China; ^3^Department of Developmental and Behavioral Pediatrics of Shanghai Children's Medical Center, XinHua Hospital Affiliated to Shanghai Jiao Tong University School of Medicine, Shanghai Key Laboratory of Children's Environmental Health, Shanghai 200127, China

## Abstract

Primary monosymptomatic nocturnal enuresis (PMNE) is a common developmental disorder in children. Previous literature has suggested that PMNE not only is a micturition disorder but also is characterized by cerebral structure abnormalities and dysfunction. However, the biological mechanisms underlying the disease are not thoroughly understood. Graph theoretical analysis has provided a unique tool to reveal the intrinsic attributes of the connectivity patterns of a complex network from a global perspective. Resting-state fMRI was performed in 20 children with PMNE and 20 healthy controls. Brain networks were constructed by computing Pearson's correlations for blood oxygenation level-dependent temporal fluctuations among the 2 groups, followed by graph-based network analyses. The functional brain networks in the PMNE patients were characterized by a significantly lower clustering coefficient, global and local efficiency, and higher characteristic path length compared with controls. PMNE patients also showed a reduced nodal efficiency in the bilateral calcarine sulcus, bilateral cuneus, bilateral lingual gyri, and right superior temporal gyrus. Our findings suggest that PMNE includes brain network alterations that may affect global communication and integration.

## 1. Introduction

Nocturnal enuresis is a common developmental disorder that affects 15–20% of 5-year-old children [1]. This disorder can persist in adolescence and has important negative effects on the self-image and performance of these children [2]. The condition is defined as primary monosymptomatic nocturnal enuresis (PMNE) when a child has enuresis without additional lower urinary tract symptoms (excluding nocturia) or a history of bladder dysfunction and has never had a period of established urinary continence for more than six months [3].

Several factors are associated with and contribute to nocturnal enuresis, including heredity, polyuria, detrusor overactivity, sleep, and central nervous system mechanisms [4]. Recently, maturational delays of the central nervous system have been indicators of PMNE pathogenesis. Toros et al. reported an increased frequency of a high-level hyperventilation response in recordings of a resting-state electroencephalogram, suggesting the existence of delayed cortical maturity in PMNE [5]. Event-related brain potentials have also been used to study enuresis; results have shown longer P300 latency in primary enuretics compared with nonenuretics [6], and P300 amplitude is decreased in the parietal recordings of enuretics when compared with the controls [7], which is evidence of a maturational delay in the central nervous system function [6, 7]. Freitag et al. also reported the existence of increased I-III and I-V interpeak latencies of the brainstem auditory evoked potential, suggesting maturational deficits in the brain stems of nocturnal enuretic children [8]. Desamino-arginine vasopressin and alarm therapy for nocturnal enuresis were shown to increase the prepulse inhibition of startle reflexes, thus supporting the hypothesis of a maturational delay of reflex inhibition in nocturnal enuresis [9, 10].

In recent years, magnetic resonance imaging (MRI) techniques, such as structural MRI, functional MRI, and diffusion MRI, have provided an efficient, feasible, and noninvasive method to investigate the biological mechanisms of incontinence. Using functional MRI, a number of studies have reported alterations in several brain functions in patients with urgency and urge incontinence [11, 12]. One study utilized event-related fMRI in PMNE subjects and revealed that children with PMNE had deficits in working memory [13]. Abnormal functional connectivity has also been found in children with PMNE, including the cerebellum, frontal lobe, and thalamus in the cerebello-thalamo-frontal circuit [14]. We have performed a series of MRI experiments to investigate the functional and structural abnormalities that are associated with PMNE. We previously reported that forebrain activation was altered during a response inhibition task [15] and that the nature of the local intrinsic activity (i.e., amplitude low frequency fluctuation and regional homogeneity) changed in the prefrontal cortex during the resting state in children with PMNE [16]. We also identified microstructural abnormalities in the thalamus, the medial frontal gyrus, the anterior cingulated cortex, and the insular cortex of children with PMNE using diffusion tensor imaging [17] and neurochemical abnormalities in the prefrontal cortex and the pons of children with PMNE using proton magnetic resonance spectroscopy [18]. These studies showed that children with PMNE have structural, functional, and neurochemical abnormalities in the brain.

Prior studies mostly focused on several regions which involved cerebral micturition control network. In fact, children with PMNE probably have more serious problems beyond the micturition control; they probably have potential cognitive problems, such as working memory [14] and response inhibition [15]. Actually, the whole brain can be modeled as a large-scale complex network; its function can be fulfilled through both segregated and integrated specific functional connections patterns with optimized efficiency [19, 20]. The investigation of PMNE-related alterations in whole brain functional networks, instead of specific brain regions or local networks, may give further network-level information about the children with PMNE. Up to now, little is known about the PMNE-related alterations in topological properties, especially the topological efficiency of the whole brain functional networks during resting state. The advantage of a graph theory-based network analysis is that it provides measures for both global and local connectivity.

The topological organization of brain networks has recently been studied with graph theory [19, 21, 22]. Graph theory-based approaches model the brain as a complex network, representing it graphically using a collection of nodes and edges. Generally speaking, a network consists of *N* nodes that are linked by *K* edges. Networks can be described by an adjacent matrix *A*(*n*, *n*) in which *n* is the number of nodes and the value of *A*_*ij*_ refers to the edge linking node *i* and node *j*. There are many graph metrics that can be used to describe the topological properties of a network, including the clustering coefficient (*C*_*p*_), characteristic path length (*L*_*p*_), normalized clustering coefficient (*γ*), normalized characteristic path length (*λ*), small-worldness (*σ*), global efficiency (*E*_glob_), local efficiency (*E*_loc_), nodal betweenness, nodal degree, and nodal efficiency. After the network modeling procedure, various graph theoretical metrics can be used to investigate the organizational mechanism underlying the relevant networks. The graph-based network analyses enable us not only to visualize the overall connectivity pattern among all brain regions but also to quantitatively characterize the global organization. Graph-based techniques used to study brain networks, including normal development, aging, and various brain disorders, have increased [21–25]. Previous studies showed that the brain's intrinsic activity is organized as a small-world, highly efficient network, with significant modularity and highly connected hub regions [19], which have also been found to change throughout normal development and aging and in various pathological conditions [21–25]. In the present paper, we propose a connectome-scale assessment of functional connectivity for children with PMNE via resting-state fMRI data.

## 2. Methods

### 2.1. Subjects

We studied 24 children with PMNE and 29 healthy children with the consent of the children and their guardians. All children were right-handed with an IQ greater than 75, and the presence of neurological and psychiatric diseases was excluded based on both a clinical examination (the DSM-IV criteria) and a structured interview. All children with PMNE were outpatients of the Shanghai Children's Medical Center. This study was approved by the IRB of the Shanghai Children's Medical Center in affiliation with the Shanghai Jiao Tong University School of Medicine (number SCMC-201014).

The inclusion criteria for all participants were listed as follows: (1) a physical, psychiatric, and neurological evaluation conducted by at least 3 members of a team of certified and experienced developmental and behavioral pediatricians; (2) age 7–15 years; (3) right-handedness; (4) children diagnosed with PMNE based on the criteria: a child has enuresis without additional lower urinary tract symptoms (excluding nocturia) or history of bladder dysfunction and has never had a period of established urinary continence for more than six months; (5) the Wechsler Intelligence Scale for Children-Revised (WISC-R) test employed to determine the intelligence quotient (IQ) of all subjects; and (6) the number and gender of each subgroup being matched.

The exclusion criteria for this study were listed as follows: (1) attention deficit/hyperactivity disorder, autism, or any psychiatric comorbid disorders; (2) previous head trauma, neurologic disorders, psychosurgery, or substantial physical illness; (3) fMRI data with obvious artifacts and distortions; (4) left-handedness, as assessed with the Annett Hand Preference Questionnaire; and (5) a full-scale IQ less than 80 according to an age appropriate WISC-Chinese Revision.

Due to excessive head motion in some cases, functional images of 20 PMNE patients and 20 healthy children were available for further analysis. There were 2 groups of children: the average age was 10.8 ± 2.0 years for the PMNE group (14 males, 6 females) and 9.9 ± 2.0 years for the normal control group (14 males, 6 females). Additional clinical data regarding the patient groups are shown in Supplementary Material 1 in Supplementary Material available online at http://dx.doi.org/10.1155/2015/463708.

### 2.2. Data Acquisition

All subjects underwent a resting-state functional MRI scan using a 3T magnetic resonance system (Siemens, Magnetom Trio Tim) with a 12-channel phased array head coil. The sequence parameters were as follows: repetition time/echo time (*T*_*R*_/*T*_*E*_) = 2,000/30 ms; flip angle = 90°; 32 axial slices per volume; 3 mm slice thickness (33% dist. factor); matrix = 64 × 64; and FOV = 220 × 220 mm^2^. Each functional run contained 210 image volumes, resulting in a total scanning time of 420 s for each participant. The first ten scans were discarded before the preprocessing of the data to remove the impact of magnetization stabilization. All participants were instructed not to focus their thoughts on anything in particular and to keep their eyes closed during the acquisition.

### 2.3. Data Preprocessing

Image preprocessing was performed using the SPM8 package (http://www.fil.ion.ucl.ac.uk/spm/; Wellcome Trust Centre for Neuroimaging, University College London, United Kingdom) and Graph Theoretical Network Analysis (GRETNA, http://www.nitrc.org/projects/gretna/). First, for each participant, the first 10 time points were discarded to avoid the instability of the initial MRI signal and to familiarize the subjects with the fMRI scanning noise. Next, the remaining fMRI data were corrected for the intravolume acquisition time delay and head motion. The head motion parameters of all participants were determined, and the extendable inclusion criteria for translational movement were <3.0 mm and <3.0° rotation. After these corrections, the images were spatially normalized to the standard space of the standard Montreal Neurological Institute (MNI) template by applying the EPI template at a 3 × 3 × 3 mm^3^ resolution. Finally, the resulting data were further filtered through a temporal band pass (0.0–0.08 Hz) to reduce the effects of low-frequency drift and high-frequency physiological noises.

### 2.4. Network Construction and Analysis

We used GRETNA to construct the network. To define the brain nodes, an atlas of Automated Anatomical Labeling [26] was employed to divide the entire brain into 90 cortical and subcortical regions of interest, each representing a node of the network. To define the network edges, first, the representative mean time series of each region was acquired by averaging the time series of all voxels within that region, followed by a correction of head motion effects by regressing out the head motion profiles estimated in the image realignment from the mean time-course. Then, the residuals of the regression analyses were used to compute the partial correlation in this study, resulting in a 90 × 90 partial correlation matrix for each subject. Finally, individual partial correlation matrices were converted into weighted matrices; this method has been used in previous brain network studies [27–31].

We applied a wide range sparsity threshold *S* to all correlation matrices, which is determined in this procedure to guarantee that the threshold networks were estimable for small-worldness (scalar *σ* was larger than 1.1) and had sparse properties with as few spurious edges as possible and the average degree over all nodes of each threshold network was larger than 2 × log⁡(90) [29, 32]. Our generated threshold range was 0.10 < *S* < 0.34 with an interval of 0.01. For brain networks at each sparsity threshold, we calculated both global and node network metrics.

The global metrics included (1) small-world parameters [32] (i.e., clustering coefficient *C*_*p*_, characteristic path length *L*_*p*_, normalized clustering coefficient *γ*, normalized characteristic path length *λ*, and small-worldness *σ*) and (2) network efficiency [33] (i.e., local efficiency *E*_loc_ and global efficiency *E*_glob_). The node metrics induced three nodal centrality metrics: the degree, efficiency, and betweenness. Furthermore, we calculated the area under the curve (AUC) for each network metric, which provides a summarized scalar for the topological characterization of brain networks independent of a single threshold selection. The AUC for a network metric *Y*, which was calculated over the sparsity threshold range of *S*_1_ to *S*_*n*_ with interval of Δ*S*, was computed as *Y* ^AUC^ = ∑_*k*=1_^*n*−1^[*Y*(*S*_*k*_) + *Y*(*S*_*k*+1_)] × Δ*S*/2. In the current study, *S*_1_ = 0.10, *S*_*n*_ = 0.34, and Δ*S* = 0.01 (supplementary materials 2). The AUC metric has been used in previous brain network studies and is sensitive in detecting topological alterations of brain disorders [29, 34–36].

Moreover, to further localize the specific pairs of brain regions in which functional connectivity was altered in the PMNE patients, we identified region pairs that exhibited between-group differences in nodal characteristics and utilized the network based statistics (NBS) method (http://www.nitrc.org/projects/nbs/) [37] to localize the connected networks that exhibited significant changes in the PMNE patients.

Specially, we firstly choose the nodes which exhibited between-group differences in at least one of the three nodal centralities (the node degree, efficiency, and betweenness). Then a subset of connections matrix for each participant was conducted according to these altered nodes. Subsequently, the NBS approach was applied to define a set of suprathreshold links that included any connected components (threshold, *T* = 1.6, *P* < 0.05). To estimate the significance for each component, the nonparametric permutation approach (10000 permutations) was also conducted. For a detailed description, see [37].

### 2.5. Statistical Analysis

We used nonparametric permutation tests [29] for each network metric's AUC. We compared the overall topologies (i.e., small-world properties, weight clustering coefficient, and weight characteristic shortest path length) and the nodal characteristics (i.e., nodal degree, nodal efficiency, and betweenness centrality) of the functional connectivity network between the patients and controls. Briefly, we first calculated the between-group difference in the mean value of each network metric. To test the null hypothesis that the observed group differences could occur by chance, for each network metric we then randomly reallocated all the values into two groups and recomputed the mean differences between the two randomized groups. This randomization procedure was repeated 10,000 times, and the 95th percentile points of each distribution were used as the critical values for a two-tailed test of the null hypothesis with a probability of type I error of 0.05. Additionally, to address the problem of multiple comparisons, the nodal centralities were tested on whether they survived a false discovery rate (FDR) threshold of *q* = 0.05.

## 3. Results

### 3.1. Global Topological Organization of the Functional Connectome

**Figure 1:**
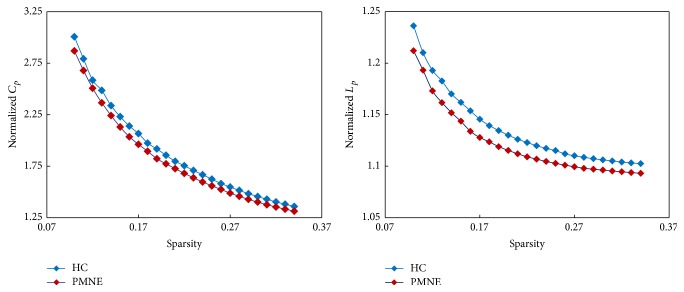
The key small-world parameters of functional network as a function of sparsity threshold. Both PMNE group and non-PMNE group showed normalized *C*_*p*_ larger than 1 and normalized *L*_*p*_ approximately equal to 1, indicating both groups exhibited a small-world topology. PMNE: children with primary monosymptomatic nocturnal enuresis; HC: healthy children; *C*_*p*_: clustering coefficient; *L*_*p*_: characteristic path length.

The whole-brain connectome of both the PMNE and control groups exhibited typical features of small-world topology; that is, compared with matched random networks, the functional brain networks had higher clustering coefficients (*C*_*p*_) but similar characteristic path length (*L*_*p*_) (Figure 1).

**Figure 2:**
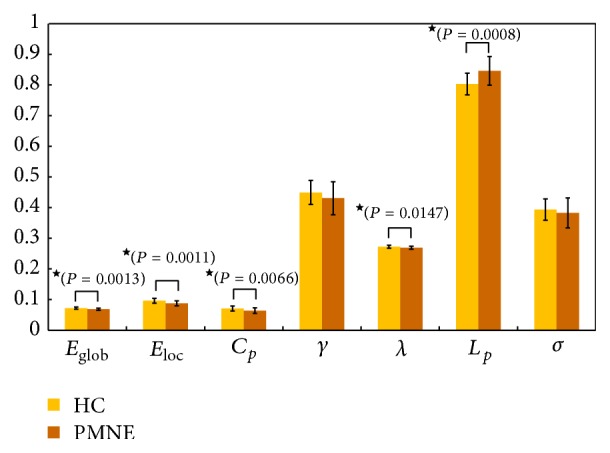
Differences in topological properties of functional brain networks between pediatric PMNE patients and trauma exposed non-PMNE controls. Significant differences were found in *C*_*p*_ (*P* = 0.0066), *λ* (*P* = 0.0147), *E*_glob_ (*P* = 0.0013), and *E*_loc_ (*P* = 0.0011) between pediatric PMNE patients and non-PMNE controls. ★: the black stars indicate the significantly statistical difference between the two groups (*P* < 0.05, uncorrected). Error bars denote standard deviations. PMNE: children with primary monosymptomatic nocturnal enuresis; HC: healthy children; *E*_glob_: global efficiency; *E*_loc_: local efficiency; *C*_*p*_: clustering coefficient; *γ*: normalized clustering coefficient; *λ*: normalized characteristic path length; *L*_*p*_: characteristic path length; *σ*: small-worldness.

The patient group showed significantly lower values for *C*_*p*_ (*P* = 0.007) and higher values for *L*_*p*_ (*P* = 0.0008). No significant (*P* > 0.05) differences were found in the *λ*, *γ*, or *σ*. In terms of network efficiency, the comparisons revealed significant decreases in both *E*_glob_ (*P* = 0.001) and *E*_loc_ (*P* = 0.001) in the functional brain networks of the patients compared with the healthy controls (Figure 2).

### 3.2. Regional Topological Organization of the Functional Connectome

**Figure 3:**
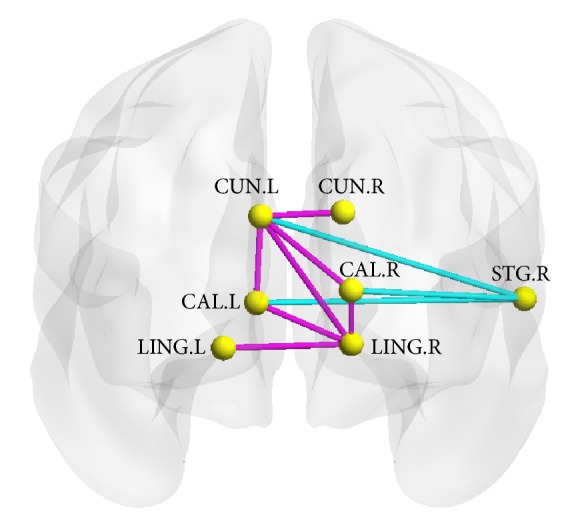
The region pairs showing altered nodal centralities brain regions and functional connections in the PMNE patients. These connections formed a single connected network with 7 nodes and 10 connections, which was significantly (*P* < 0.05, corrected) abnormal in the patients. Edge in cyan: increased functional connections in the PMNE patients; edge in magenta: decreased functional connections in the PMNE patients. CUN, cuneus; LING, lingual gyrus; CAL, calcarine sulcus; STG, superior temporal gyrus; R, right hemisphere; P, posterior. The nodes and connections were mapped onto the cortical surfaces using the BrainNet Viewer package (http://www.nitrc.org/projects/bnv).

We identified the brain regions showing significant between-group differences in at least one nodal metric (*P* < 0.05, FDR corrected). Compared with normal control subjects, the patients showed decreased nodal centralities in several brain regions, including the bilateral calcarine sulcus, the bilateral cuneus, the bilateral lingual gyri, and the right superior temporal gyrus (Figure 3, Table 1). There were no significantly increased nodal centralities.

### 3.3. Disrupted Functional Network Connectivity in the Patients

Using NBS [37], we identified a connected network with 7 nodes and 10 connections; this network was significantly altered in the PMNE group (*P* < 0.05, corrected) (Figure 3, Table 2). Within this network, both decreased and increased connections were detected in the patients compared with the control subjects. The connections of the left cuneus in the bilateral calcarine sulcus, the left cuneus in the right cuneus, and the left cuneus in the right lingual gyrus were decreased. Furthermore, the connections of the right lingual gyrus in the left lingual gyrus and the right lingual gyrus in bilateral calcarine sulcus were also decreased. On the other side, the increased connections included the right superior temporal gyrus in the bilateral calcarine sulcus and the right superior temporal gyrus in the left cuneus.

## 4. Discussion

### 4.1. Global Topological Organization of the Functional Connectome

In our study, we computed small-word metrics of 90 node networks based on the ALL template and found that *λ*, *γ*, and *σ* of the functional networks were not significantly changed in the PMNE group; however, the patient group showed significantly lower values for *C*_*p*_ and higher values for *L*_*p*_, and the network efficiency parameters *E*_glob_ and *E*_loc_ were significantly decreased in the functional brain networks of the patients.

According to graph theory, a high *C*_*p*_ indicates that the nodes tend to form dense regional cliques, implying that the efficiency in local information transfer and processing is high [38]; a low *L*_*p*_ indicates a high transfer speed through the overall network, implying that the network has a high global efficiency [38]. Thus, the *C*_*p*_ and *E*_loc_ were reduced in PMNE group, which demonstrated that the efficiency in local information transfer and processing is low in children with PMNE. The higher *L*_*p*_ and lower *E*_glob_ in the PMNE group suggested that the brain network has a low global efficiency and a low ability of integrating information. Our present results are correlated with the previous findings that the efficiency of regional information processing and the efficiency of overall information transfer across the entire network were reduced in children with PMNE [38]. Therefore, our results suggest that intrinsic brain functional organization was disrupted in PMNE and that there was reduced efficiency in information exchange and integration in both local and global regions.

### 4.2. Regional Topological Organization of the Functional Connectome

Children with PMNE showed significant decreased nodal efficiency in the bilateral calcarine sulcus, bilateral cuneus, bilateral lingual gyri, and right superior temporal gyrus. The calcarine sulcus, cuneus, and lingual gyrus are in the occipital lobe, which is part of visual network. The right superior temporal gyrus is an essential structure involved in auditory processing and part of the auditory network.

Tomasi and Volkow reported that the cuneus is one of 4 major cortical hubs, and the cuneus hub network is both correlated with the somatosensory network and anticorrelated with the default mode network activity of other networks [39]. The calcarine cortex, lingual gyri, fusiform, occipital gyri, and paracentral lobule were the secondary hubs identified in this network, which suggests that the visual processing performed by the secondary hubs is integrated in the cuneus [39]. A previous study suggests that the cuneus and lingual gyrus take part in stop-down processes of visuospatial attention [40]. The cuneus is an important node of the default mode network and is also affected by attention-deficit/hyperactivity disorder [41, 42]. Decreased connectivity in the left cuneus has also been found in individuals with borderline personality disorder [43]. Additionally, gray matter volume in the cuneus has been suggested to be associated with better inhibitory control in bipolar depression patients [44], and dysfunction has been reported in the response inhibition in children with PMNE [15]. Thus, reduced nodal efficiency in the cuneus may point to insufficiencies in communications between executive control regions and visual processing regions.

In our results, the nodal efficiency was reduced in the bilateral lingual gyri, and the degree was reduced in the right lingual gyrus. Interestingly, the lingual gyrus has been associated with psychopathology, such as major depression [45], posttraumatic stress disorder [46, 47], and childhood maltreatment [48]. A previous task related neuroimaging study reported that the lingual gyrus was associated with the identification of facial expressions of emotion [49]. Equit et al. reported that children with nocturnal enuresis processed emotions differently from children with attention-deficit/hyperactivity disorder and controls [50]. Thus, we speculated that the abnormality in the lingual gyri would be related to the burden of disease and negative psychological factors.

### 4.3. Disrupted Functional Network Connectivity in the Patients

The NBS method analysis showed increased connections between auditory system (right superior temporal gyrus) and visual system and decreased connections within the visual network, including the bilateral calcarine sulcus, the bilateral cuneus, and the bilateral lingual gyri. These findings suggest that connectivity of the auditory and visual networks is unbalanced in children with PMNE.

Visual information is the main source of information, and the visual network is closely linked to other brain networks. When the visual network functions abnormally, a great potential for harm is induced, possibly resulting in damage to cognitive function. Abnormal visual network functionality can also result in reduced efficiency of the global network and can affect global communication and integration. The network efficiency of both *E*_glob_ and *E*_loc_ in the functional brain networks of the patients was significantly decreased. This observation suggests that the disruption of brain communication and integration may also be an important factor in PMNE disorders.

Several issues must be addressed further. First, the brain networks were constructed by parcellating the entire brain into 90 brain regions at a coarsely regional level. Future studies should use a more precise parcellation strategy or spatial scale to overcome this challenge. Second, the functional brain networks constructed from the r-fMRI data were largely constrained by anatomical pathways [51, 52]. Thus, combining multimodal neuroimaging data could facilitate the uncovering of the structure-function relationships in PMNE patients. Finally, the results in this paper are different from those of our previous resting-state fMRI study [16], which focused on local intrinsic activity. We did not identify a direct relationship between the results in this study and the pathology of PMNE. However, this study sheds light on the functional connectivity of children with PMNE and suggests that there may be potential cognition dysfunction in children with PMNE.

It is important to note some limitations in this study. First, functional brain networks were constructed at a regional level by parcellating the whole brain into 90 regions based on a previously published atlas. Brain networks derived using different parcellation schemes or at different spatial scales exhibit distinct topological architectures [53, 54]. Further studies are needed to determine which brain parcellation strategy or spatial scale is more appropriate for the characterization of network topology in PMNE. Second, we have not found the direct relationship between our results and the pathology of PMNE. The regions showing abnormality nodal centralities in PMNE patients were not involved in the micturition neural control network. However, our results give direct evidence that the PMNE probably has cognitive problem.

## 5. Conclusion

This is the first study to investigate the characteristics of PMNE patients with network-based graph theory using resting-state fMRI. In the PMNE group, there were reduced local and global efficiency in the brain, as well as disturbances in connectivity. The alterations in network topologies primarily occurred in the right superior temporal gyrus (auditory network), and decreased connectivity was observed in the visual network, including the bilateral calcarine sulcus, the bilateral cuneus, and the bilateral lingual gyri. Our findings suggest that PMNE includes brain network alterations, which may affect both local and global communication and integration.

## Supplementary Material

Supplementary Material 1 is the clinical data collected from the patient group, including bed-wetting frequency, bladder volume, frequency of waking up for voluntary voiding, and so on.
Supplementary Material 2: The illustration of area under curve (AUC) for network metrics.

## Figures and Tables

**Table 1:** Regions showing decreased nodal centralities in PMNE patients as compared with control subjects.

Brain regions	*P* values		
	Nodal betweenness	Nodal degree	Nodal efficiency
Left calcarine sulcus	0.460	0.016	**0.001**
Right calcarine sulcus	0.383	0.023	**0.002**
Left cuneus	0.124	0.022	**0.001**
Right cuneus	0.502	0.017	**0.002**
Left lingual gyrus	0.351	0.008	**0.000**
Right lingual gyrus	0.128	**0.000**	**0.000**
Right superior temporal gyrus	0.020	0.017	**0.003**

Regions were considered abnormal in PMNE patients if they exhibited significant between-group differences (FDR corrected *P* < 0.05 shown in bold font) in at least one of the three nodal centralities.

**Table 2:** Altered functional connections in the PMNE patients group as compared to the control group.

Region 1	Region 2	*t*-score	Increase/decrease
Right superior temporal gyrus	Left cuneus	3.95	Increase
Right superior temporal gyrus	Left calcarine sulcus	2.29	Increase
Right superior temporal gyrus	Right calcarine sulcus	2.21	Increase
Left cuneus	Left calcarine sulcus	2.25	Decrease
Left cuneus	Right cuneus	1.75	Decrease
Left cuneus	Right calcarine sulcus	1.70	Decrease
Left cuneus	Right lingual gyrus	1.65	Decrease
Right lingual gyrus	Left lingual gyrus	3.36	Decrease
Right lingual gyrus	Left calcarine sulcus	2.06	Decrease
Right lingual gyrus	Right calcarine sulcus	1.72	Decrease

Connections are listed in descending order of statistical significance (*P*< 0.05). These connections formed a connected network that was identified using a network-based statistical approach. See Figure 2 for a graphical representation of these connections.

## References

[1] Riccabona M. (2010). Evaluation und management of enuresis: an update. *Urologe—Ausgabe A*.

[2] Theunis M., van Hoecke E., Paesbrugge S., Hoebeke P., Vande Walle J. (2002). Self-image and performance in children with nocturnal enuresis. *European Urology*.

[3] Nevéus T., von Gontard A., Hoebeke P. (2006). The standardization of terminology of lower urinary tract function in children and adolescents: report from the Standardisation Committee of the International Children's Continence Society. *Journal of Urology*.

[4] Nevéus T. (2009). Diagnosis and management of nocturnal enuresis. *Current Opinion in Pediatrics*.

[5] Toros F., Özge A., Bozlu M., Çayan S. (2003). Hyperventilation response in the electroencephalogram and psychiatric problems in children with primary monosymptomatic nocturnal enuresis. *Scandinavian Journal of Urology and Nephrology*.

[6] Iscan A., Ozkul Y., Unal D. (2002). Abnormalities in event-related potential and brainstem auditory evoked response in children with nocturnal enuresis. *Brain and Development*.

[7] Karlidag R., Ozisik H. I., Soylu A. (2004). Topographic abnormalities in event-related potentials in children with monosyptomatic nocturnal enuresis. *Neurourology and Urodynamics*.

[8] Freitag C. M., Röhling D., Seifen S., Pukrop R., von Gontard A. (2006). Neurophysiology of nocturnal enuresis: evoked potentials and prepulse inhibition of the startle reflex. *Developmental Medicine & Child Neurology*.

[9] Schulz-Juergensen S., Langguth A., Eggert P. (2014). Effect of alarm therapy on conditioning of central reflex control in nocturnal enuresis: pilot study on changes in prepulse inhibition (PPI). *Pediatric Nephrology*.

[10] Schulz-Juergensen S., Rieger M., Schaefer J., Neusuess A., Eggert P. (2007). Effect of 1-desamino-8-D-arginine vasopressin on prepulse inhibition of startle supports a central etiology of primary monosymptomatic enuresis. *Journal of Pediatrics*.

[11] Fowler C. J., Griffiths D. J. (2010). A decade of functional brain imaging applied to bladder control. *Neurourology and Urodynamics*.

[12] Griffiths D. J., Tadic S. D. (2008). Bladder control, urgency, and urge incontinence: evidence from functional brain imaging. *Neurourology and Urodynamics*.

[13] Yu B., Guo Q., Fan G., Ma H., Wang L., Liu N. (2011). Evaluation of working memory impairment in children with primary nocturnal enuresis: evidence from event-related functional magnetic resonance imaging. *Journal of Paediatrics and Child Health*.

[14] Yu B., Sun H., Ma H. (2013). Aberrant whole-brain functional connectivity and intelligence structure in children with primary nocturnal enuresis. *PLoS ONE*.

[15] Lei D., Ma J., Du X., Shen G., Tian M., Li G. (2012). Altered brain activation during response inhibition in children with primary nocturnal enuresis: an fMRI study. *Human Brain Mapping*.

[16] Lei D., Ma J., Du X., Shen G., Tian M., Li G. (2012). Spontaneous brain activity changes in children with primary monosymptomatic nocturnal enuresis: a resting-state fMRI study. *Neurourology and Urodynamics*.

[17] Lei D., Ma J., Shen X. (2012). Changes in the brain microstructure of children with primary monosymptomatic nocturnal enuresis: a diffusion tensor imaging study. *PLoS ONE*.

[18] Zhang J., Lei D., Ma J. (2014). Brain metabolite alterations in children with primary nocturnal enuresis using proton magnetic resonance spectroscopy. *Neurochemical Research*.

[19] He Y., Evans A. (2010). Graph theoretical modeling of brain connectivity. *Current Opinion in Neurology*.

[20] Bassett D. S., Bullmore E. (2006). Small-world brain networks. *Neuroscientist*.

[21] Wang J., Zuo X., He Y. (2010). Graph-based network analysis of resting-state functional MRI. *Frontiers in Systems Neuroscience*.

[22] Iturria-Medina Y. (2013). Anatomical brain networks on the prediction of abnormal brain states. *Brain Connectivity*.

[23] Wu K., Taki Y., Sato K. (2013). Topological organization of functional brain networks in healthy children: differences in relation to age, sex, and intelligence. *PLoS ONE*.

[24] Cao M., Wang J.-H., Dai Z.-J. (2014). Topological organization of the human brain functional connectome across the lifespan. *Developmental Cognitive Neuroscience*.

[25] Tijms B. M., Wink A. M., de Haan W. (2013). Alzheimer's disease: connecting findings from graph theoretical studies of brain networks. *Neurobiology of Aging*.

[26] Tzourio-Mazoyer N., Landeau B., Papathanassiou D. (2002). Automated anatomical labeling of activations in SPM using a macroscopic anatomical parcellation of the MNI MRI single-subject brain. *NeuroImage*.

[27] Liu Y., Liang M., Zhou Y. (2008). Disrupted small-world networks in schizophrenia. *Brain*.

[28] Ferrarini L., Veer I. M., Baerends E. (2009). Hierarchical functional modularity in the resting-state human brain. *Human Brain Mapping*.

[29] Zhang J., Wang J., Wu Q. (2011). Disrupted brain connectivity networks in drug-naive, first-episode major depressive disorder. *Biological Psychiatry*.

[30] Salvador R., Suckling J., Coleman M. R., Pickard J. D., Menon D., Bullmore E. (2005). Neurophysiological architecture of functional magnetic resonance images of human brain. *Cerebral Cortex*.

[31] He Y., Chen Z., Evans A. (2008). Structural insights into aberrant topological patterns of large-scale cortical networks in Alzheimer's disease. *The Journal of Neuroscience*.

[32] Watts D. J., Strogatz S. H. (1998). Collective dynamics of “small-world” networks. *Nature*.

[33] Latora V., Marchiori M. (2001). Efficient behavior of small-world networks. *Physical Review Letters*.

[34] Achard S., Bullmore E. (2007). Efficiency and cost of economical brain functional networks. *PLoS Computational Biology*.

[35] He Y., Dagher A., Chen Z. (2009). Impaired small-world efficiency in structural cortical networks in multiple sclerosis associated with white matter lesion load. *Brain*.

[36] Wang J., Wang L., Zang Y. (2009). Parcellation-dependent small-world brain functional networks: a resting-state fmri study. *Human Brain Mapping*.

[37] Zalesky A., Fornito A., Bullmore E. T. (2010). Network-based statistic: identifying differences in brain networks. *NeuroImage*.

[38] Xie T., He Y. (2012). Mapping the alzheimer's brain with connectomics. *Frontiers in Psychiatry*.

[39] Tomasi D., Volkow N. D. (2011). Association between functional connectivity hubs and brain networks. *Cerebral Cortex*.

[40] Hahn B., Ross T. J., Stein E. A. (2006). Neuroanatomical dissociation between bottom-up and top-down processes of visuospatial selective attention. *NeuroImage*.

[41] de Celis Alonso B., Tobón S. H., Suarez P. D. (2014). A multi-methodological MR resting state network analysis to assess the changes in brain physiology of children with ADHD. *PLoS ONE*.

[42] Wang X., Jiao Y., Tang T., Wang H., Lu Z. (2013). Altered regional homogeneity patterns in adults with attention-deficit hyperactivity disorder. *European Journal of Radiology*.

[43] Wolf R. C., Sambataro F., Vasic N. (2011). Aberrant connectivity of resting-state networks in borderline personality disorder. *Journal of Psychiatry and Neuroscience*.

[44] Haldane M., Cunningham G., Androutsos C., Frangou S. (2008). Structural brain correlates of response inhibition in Bipolar Disorder I. *Journal of Psychopharmacology*.

[45] Veer I. M., Beckmann C. F., van Tol M.-J. (2010). Whole brain resting-state analysis reveals decreased functional connectivity in major depression. *Frontiers in Systems Neuroscience*.

[46] Qin L.-D., Wang Z., Sun Y.-W. (2012). A preliminary study of alterations in default network connectivity in post-traumatic stress disorder patients following recent trauma. *Brain Research*.

[47] Yin Y., Jin C., Eyler L. T., Jin H., Hu X., Duan L. (2012). Altered regional homogeneity in post-traumatic stress disorder: a resting-state functional magnetic resonance imaging study. *Neuroscience Bulletin*.

[48] van der Werff S. J. A., Pannekoek J. N., Veer I. M. (2013). Resilience to childhood maltreatment is associated with increased resting-state functional connectivity of the salience network with the lingual gyrus. *Child Abuse and Neglect*.

[49] Kitada R., Johnsrude I. S., Kochiyama T., Lederman S. J. (2010). Brain networks involved in haptic and visual identification of facial expressions of emotion: an fMRI study. *NeuroImage*.

[50] Equit M., Becker A., El Khatib D., Rubly M., Becker N., von Gontard A. (2014). Central nervous system processing of emotions in children with nocturnal enuresis and attention-deficit/hyperactivity disorder. *Acta Paediatrica*.

[51] Damoiseaux J. S., Greicius M. D. (2009). Greater than the sum of its parts: a review of studies combining structural connectivity and resting-state functional connectivity. *Brain Structure and Function*.

[52] Honey C. J., Sporns O., Cammoun L. (2009). Predicting human resting-state functional connectivity from structural connectivity. *Proceedings of the National Academy of Sciences of the United States of America*.

[53] Fornito A., Zalesky A., Bullmore E. T. (2010). Network scaling effects in graph analytic studies of human resting-state fMRI data. *Frontiers in Systems Neuroscience*.

[54] Zalesky A., Fornito A., Harding I. H. (2010). Whole-brain anatomical networks: does the choice of nodes matter?. *NeuroImage*.

