# Supplementary material for: Connectome-Scale Assessments of Functional Connectivity in Children with Primary Monosymptomatic Nocturnal Enuresis
Source: Biomed Res Int. 2015 Jun 9;2015:463708. doi: 10.1155/2015/463708 (PMC4477104; doi:10.1155/2015/463708)

Supplementary materials 2: The illustration of area under curve (AUC) for network metrics. The AUC for a network metric Y which was calculated over the sparisty threshold range of S1 to Sn with interval of △S, was computed as $Y^{AUC} = \sum_{k=1}^{n-1}[Y(S_k) + Y(S_{k+1})] \times \Delta S/2$. In the current study, S1= 0.10, Sn = 0.34 and △S = 0.01(Figure S1).

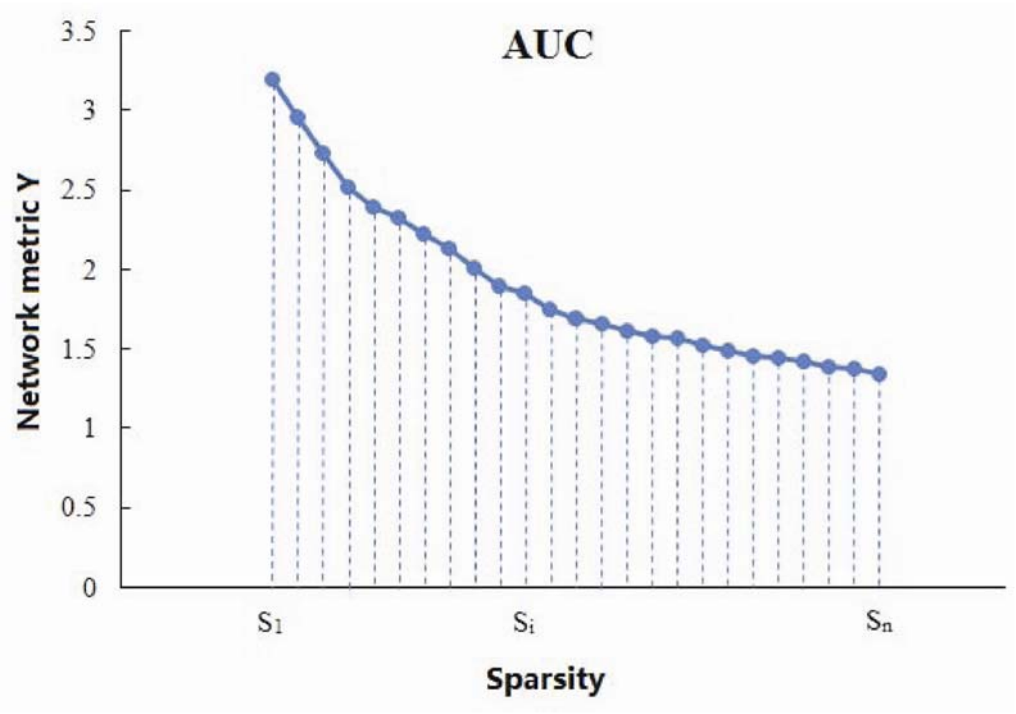

Supplement: Supplementary file 1 — Supplementary Material 1 is the clinical data collected from the patient group, including bed-wetting frequency, bladder volume, frequency of waking up for voluntary voiding, and so on. Supplementary Material 2: The illustration of area under curve (AUC) for network metrics. [file 463708.f1.zip › suppl 2.pdf]
