# Supplementary material for: Connectome-Scale Assessments of Functional Connectivity in Children with Primary Monosymptomatic Nocturnal Enuresis
Source: Biomed Res Int. 2015 Jun 9;2015:463708. doi: 10.1155/2015/463708 (PMC4477104; doi:10.1155/2015/463708)
Supplement: Supplementary file 1 — Supplementary Material 1 is the clinical data collected from the patient group, including bed-wetting frequency, bladder volume, frequency of waking up for voluntary voiding, and so on. Supplementary Material 2: The illustration of area under curve (AUC) for network metrics. [file 463708.f1.zip › suppl 1.doc]

**Supplementary material 1: Clinical data collected from the patient group.**

|  | Age (years) | Gender | Weight (kg) | Bed-wetting frequency (per week) | Bladder volume ^b^  (ml) | Frequency of waking up for voluntary voiding |
| --- | --- | --- | --- | --- | --- | --- |
| 1 | 7 | F | 25 | 4 | 122 | Sometimes |
| 2 | 8 | M | 35 | 1 | 100 | Often |
| 3 | 8 | F | 25 | 7 | 140 | Sometimes |
| 4^a^ | 8 | M | 34 | 5-6 | 260 | Sometimes |
| 5 | 8 | M | 26 | 7 | 190 | Sometimes |
| 6 | 9 | M | 48 | 1 | 130 | Sometimes |
| 7 | 10 | M | 29 | 7 | 100 | Sometimes |
| 8 | 10 | F | 33 | 4 | 205 | Sometimes |
| 9^a^ | 10 | M | 30 | 1-2 | 303 | Sometimes |
| 10^a^ | 10 | M | 30 | 2 | 130 | Sometimes |
| 11 | 10 | M | 49 | 2-3 | 155 | Sometimes |
| 12 | 11 | M | 50 | 2 | 210 | Never |
| 13 | 11 | M | 37 | 6 | 120 | Sometimes |
| 14^a^ | 11 | F | 35 | 4 | 132 | Sometimes |
| 15^a^ | 12 | M | 32 | 5 | 350 | Sometimes |
| 16^a^ | 13 | M | 41 | 2-3 | 245 | Often |
| 17 | 14 | M | 75 | 2 | 114 | Never |
| 18 | 15 | F | 50 | 7 | 510 | Never |
| 19^a^ | 15 | F | 45 | 1 | 450 | Often |
| 20 | 15 | M | 54 | 7 | 192 | Never |

All patients wet the bed only at night. The age is given in years; M = male; F = female.

^a^These children can wake up after bed-wetting.

^b^The bladder volume was defined by b-ultrasound results: children with PMNE were asked to try their best to hold back urine for three times while their bladder volumes were measured by b-ultrasound. The largest measured value was defined as the bladder volume.
